# A combined spatial score of granzyme B and CD68 surpasses CD8 as an independent prognostic factor in TNM stage II colorectal cancer

**DOI:** 10.1186/s12885-022-10048-x

**Published:** 2022-09-16

**Authors:** Luca Noti, José A. Galván, Heather Dawson, Alessandro Lugli, Richard Kirsch, Naziheh Assarzadegan, David Messenger, Philippe Krebs, Martin D. Berger, Inti Zlobec

**Affiliations:** 1 Institute of Pathology, University of Bern, Murtenstrasse 31, CH-3008 Bern, Switzerland; 2 Division of Pathology and Lab Medicine, University of Toronto, Toronto, Canada; 3 Department of Pathology, University of Michigan, Ann Arbor, USA; 4 Department of Coloproctology, University Hospitals Bristol and Weston NHS Foundation Trust, Bristol, UK; 5 Department of Medical Oncology, Bern University Hospital, Bern, Switzerland

**Keywords:** Colorectal cancer, Tumour microenvironment, Tumour immune surveillance, Digital pathology

## Abstract

**Background:**

Previous assessments of peritumoral inflammatory infiltrate in colorectal cancer (CRC) have focused on the role of CD8^+^ T lymphocytes. We sought to compare the prognostic value of CD8 with downstream indicators of active immune cell function, specifically granzyme B (GZMB) and CD68 in the tumour microenvironment.

**Methods:**

Immunohistochemical (IHC) staining was performed for CD8, GZMB, CD68 and CD163 on next-generation tissue microarrays (ngTMAs) in a primary cohort (*n* = 107) and a TNM stage II validation cohort (*n* = 151). Using digital image analysis, frequency of distinct immune cell types was calculated for tumour proximity (TP) zones with varying radii (10 μm-100 μm) around tumour cells.

**Results:**

Associations notably of advanced TNM stage were observed for low density of CD8 (*p* = 0.002), GZMB (*p* < 0.001), CD68 (*p* = 0.034) and CD163 (*p* = 0.011) in the primary cohort. In the validation cohort only low GZMB (*p* = 0.036) was associated with pT4 stage. Survival analysis showed strongest prognostic effects in the TP25μm zone at the tumour centre for CD8_,_ GZMB and CD68 (all *p* < 0.001) in the primary cohort and for CD8 (*p* = 0.072), GZMB (*p* = 0.035) and CD68 (*p* = 0.004) in the validation cohort with inferior prognostic effects observed at the tumour invasive margin. In a multivariate survival analysis, joint analysis of GZMB and CD68 was similarly prognostic to CD8 in the primary cohort (*p* = 0.007 vs. *p* = 0.002) and superior to CD8 in the validation cohort (*p* = 0.005 vs. *p* = 0.142).

**Conclusion:**

Combined high expression of GZMB and CD68 within 25 μm to tumour cells is an independent prognostic factor in CRC and of superior prognostic value to the well-established CD8 in TNM stage II cancers. Thus, assessment of antitumoral effect should consider the quality of immune activation in peritumoral inflammatory cells and their actual proximity to tumour cells.

**Supplementary Information:**

The online version contains supplementary material available at 10.1186/s12885-022-10048-x.

## Background

Colorectal cancer (CRC) is the fourth most frequent cause of death among all malignant tumours worldwide [1]. Efforts to estimate disease prognosis have led to the inclusion of numerous histopathologic tumour features in the tumour-node-metastasis (TNM) classification system [2]. However, the study of tumour characteristics in isolation provides a limited picture and does not account for the host’s immune reaction, a similarly important part of the previously proposed attacker-defender model [3]. The currently one-sided, tumour-centric approach of classification might contribute to the major heterogeneity in survival within the TNM stages in CRC. In recent decades, multiple efforts to stratify patients based on their antitumoral immune response have been made, initially using haematoxylin and eosin (H&E) stained tissue sections [4–6]. With new technical possibilities emerging, it has been possible to account for different immune cell types using immunohistochemistry [7, 8] (IHC) or immunofluorescence [9, 10] to visualize cellular expression profiles of distinct antigens while preserving the spatial context. Multiple studies have since explored a wide variety of immune cells in the peritumoral inflammatory infiltrate with tumour infiltrating lymphocytes (TILs) expressing CD3, CD8, CD45RO and FoxP3 being among the most frequently reported immune cell types prognostic for patients’ survival [7, 8, 11, 12]. To date, the Immunoscore [11, 13] presents the most integrative approach to quantify the antitumoral immune reaction and this scoring has shown prognostic value for multiple entities – including CRC – considering expression of CD3 and CD8 at the tumour centre and at the invasive margin.

Complementing TIL quantification, it has been observed that presence of tumour associated macrophages (TAMs) also indicates prognosis. Interestingly, contradictory results have been produced regarding the effect of TAMs on patient survival [8, 14–17]. This might be a consequence of macrophage subsets executing complementary functions: The M1-like phenotype is characterized by its phagocytic activities and ability to support a Th1 polarized immune response while the M2-like phenotype is associated with tissue repair processes, thus potentially promoting tumour growth [18]. Indeed, several recent studies have reported prognostic value of macrophage phenotype ratio, however still reporting controversial effects [10, 19, 20]. Examined markers include CD68 as a pan-macrophage marker complemented with CD86, IRF and iNOS for the M1 phenotype and CD163, CD206 and MAF for the M2 phenotype. Comparable efforts to differentiate state of activation in CD8^+^ T lymphocytes have been relatively scarce so far, with the serine protease granzyme B (GZMB) being the most common marker for activated cytotoxic T lymphocytes [21, 22].

The aim of this study was to examine the prognostic impact of the activated cytotoxic T lymphocyte subset as well as macrophage subsets in the peritumoral region and to compare it with the current benchmark of CD8^+^ T lymphocyte frequency in CRC patients [11, 13]. We hypothesized that consideration of activated, GZMB expressing T lymphocytes complemented with M1 polarized macrophages might reflect more precisely the actual state of tumour recognition and the quality of the antitumour immune response. To test this hypothesis, we examined markers of T lymphocyte activation and for phenotyping macrophage subsets in two independent cohorts of CRC patients, taking into account heterogeneity and spatial proximity of immune cells to their tumour cell counterparts.

## Materials and methods

### Patients

**Fig. 1:**
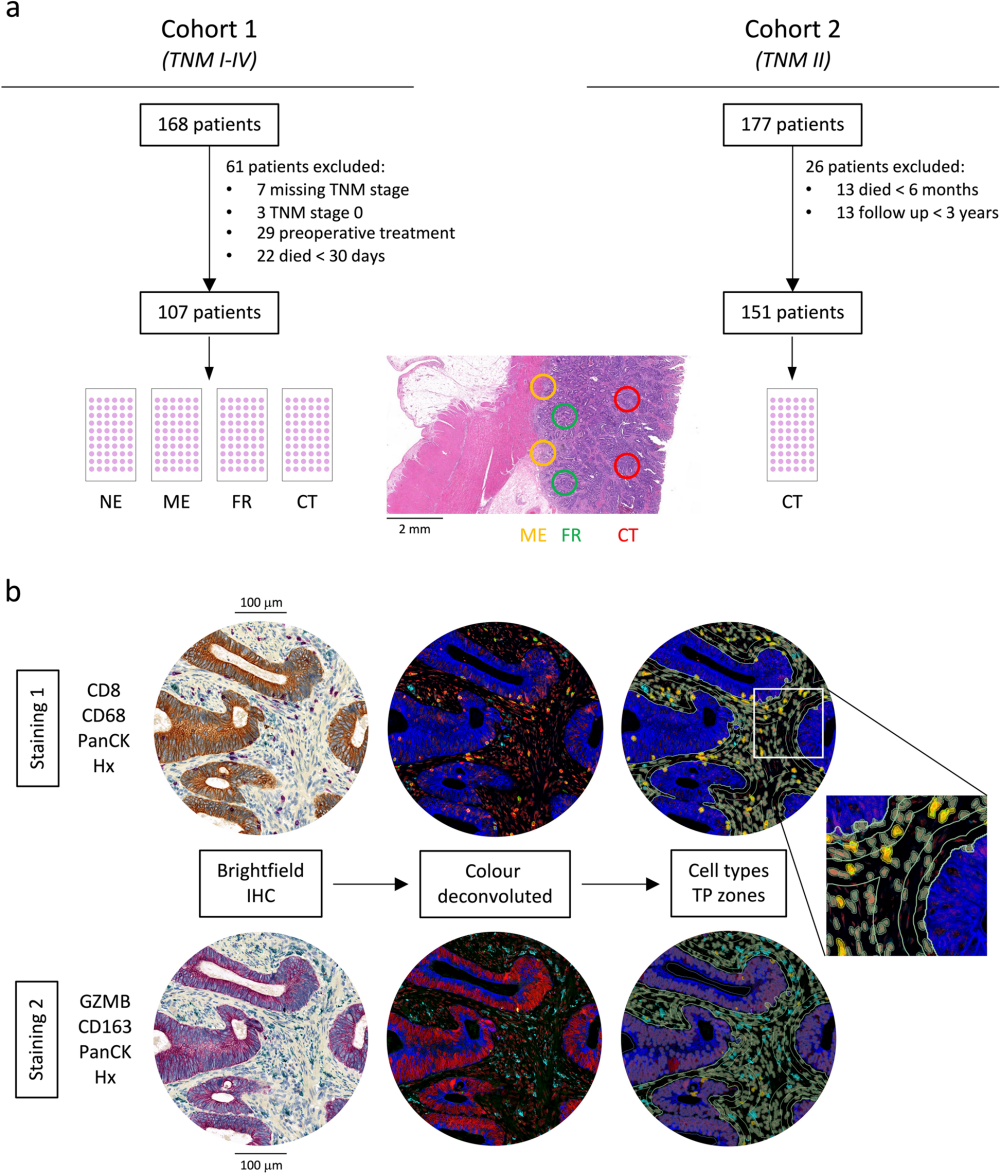
Study design. **a** shows cohort selection and distinct annotations for ngTMA® construction. **b** illustrates workflow of digital image analysis for two consecutive quadruple immunohistochemical stainings. Abbreviations: NE, normal epithelium; ME, microenvironment; FR, front; CT, centre; GZMB, granzyme B; PanCK, pancytokeratin; Hx, haematoxylin; IHC, immunohistochemistry; TP, tumour proximity

This study was conducted with two geographically independent cohorts. Cohort 1 was the primary cohort, where we intended to explore distributions, correlations, associations with clinicopathological features and prognostic significance of immune cell type densities in TNM stage I-IV patients. Subsequently, cohort 2 served as a validation cohort to confirm the findings on prognostic impact in TNM stage II patients, for whom decision to administer post-operative chemotherapy is traditionally based on tumour characteristics, leaving out host immune factors. Patient selection is shown in Fig. 1a and clinicopathological characteristics are displayed in Tables 1 and 2.

**Table 1:** Clinicopathologic patient characteristics and associations with immune cell type density in the TP25μm zone at the tumour centre in cohort 1. Tumour budding is scored according to the ITBCC criteria [23]. *Abbreviations: GZMB* granzyme B, *TBC* tumour border configuration

		CD8			GZMB			CD68		
	Overall	low (%)	high (%)	p	low (%)	high (%)	p	low (%)	high (%)	p
**n**	97	49	48		49	48		49	48	
**Age** (*n* = 97)										
Mean (SD)	69.6 (12.1)	71.3 (10.9)	67.8 (13.1)	0.162	72.1 (10.5)	67.0 (13.2)	**0.035**	69.8 (13.2)	69.3 (11.0)	0.83
**Gender** (*n* = 97)										
Female	44 (45.4)	20 (40.8)	24 (50.0)	0.481	22 (44.9)	22 (45.8)	1	19 (38.8)	25 (52.1)	0.266
Male	53 (54.6)	29 (59.2)	24 (50.0)		27 (55.1)	26 (54.2)		30 (61.2)	23 (47.9)	
**Histological Subtype** (*n* = 96)										
Adenocarcinoma	93 (96.9)	45 (93.8)	48 (100.0)	0.242	46 (95.8)	47 (97.9)	1	45 (93.8)	48 (100.0)	0.242
Mucinous	1 (1.0)	1 (2.1)	0 (0.0)		1 (2.1)	0 (0.0)		1 (2.1)	0 (0.0)	
Other	2 (2.1)	2 (4.2)	0 (0.0)		1 (2.1)	1 (2.1)		2 (4.2)	0 (0.0)	
**Location** (*n* = 85)										
Right	26 (30.6)	12 (28.6)	14 (32.6)	0.218	13 (30.2)	13 (31.0)	0.992	13 (30.2)	13 (31.0)	0.992
Left	35 (41.2)	21 (50.0)	14 (32.6)		18 (41.9)	17 (40.5)		18 (41.9)	17 (40.5)	
Rectum	24 (28.2)	9 (21.4)	15 (34.9)		12 (27.9)	12 (28.6)		12 (27.9)	12 (28.6)	
**pT** (*n* = 96)										
1	6 (6.2)	1 (2.1)	5 (10.4)	0.085	1 (2.1)	5 (10.4)	**0.036**	1 (2.1)	5 (10.4)	0.31
2	16 (16.7)	5 (10.4)	11 (22.9)		4 (8.3)	12 (25.0)		7 (14.6)	9 (18.8)	
3	52 (54.2)	28 (58.3)	24 (50.0)		30 (62.5)	22 (45.8)		27 (56.2)	25 (52.1)	
4	22 (22.9)	14 (29.2)	8 (16.7)		13 (27.1)	9 (18.8)		13 (27.1)	9 (18.8)	
**pN** (*n* = 95)										
0	61 (64.2)	22 (45.8)	39 (83.0)	**0.001**	19 (39.6)	42 (89.4)	**< 0.001**	25 (52.1)	36 (76.6)	**0.035**
1	25 (26.3)	18 (37.5)	7 (14.9)		20 (41.7)	5 (10.6)		16 (33.3)	9 (19.1)	
2	9 (9.5)	8 (16.7)	1 (2.1)		9 (18.8)	0 (0.0)		7 (14.6)	2 (4.3)	
**cM** (*n* = 97)										
0	80 (82.5)	37 (75.5)	43 (89.6)	0.12	36 (73.5)	44 (91.7)	**0.037**	37 (75.5)	43 (89.6)	0.12
1	17 (17.5)	12 (24.5)	5 (10.4)		13 (26.5)	4 (8.3)		12 (24.5)	5 (10.4)	
**TNM stage** (*n* = 97)										
I	17 (17.5)	4 (8.2)	13 (27.1)	**0.002**	3 (6.1)	14 (29.2)	**< 0.001**	5 (10.2)	12 (25.0)	**0.034**
II	38 (39.2)	15 (30.6)	23 (47.9)		15 (30.6)	23 (47.9)		16 (32.7)	22 (45.8)	
III	23 (23.7)	18 (36.7)	5 (10.4)		18 (36.7)	5 (10.4)		16 (32.7)	7 (14.6)	
IV	19 (19.6)	12 (24.5)	7 (14.6)		13 (26.5)	6 (12.5)		12 (24.5)	7 (14.6)	
**Tumour Grade** (*n* = 97)										
1	3 (3.1)	2 (4.1)	1 (2.1)	0.402	2 (4.1)	1 (2.1)	0.402	2 (4.1)	1 (2.1)	0.402
2	86 (88.7)	41 (83.7)	45 (93.8)		41 (83.7)	45 (93.8)		41 (83.7)	45 (93.8)	
3	8 (8.2)	6 (12.2)	2 (4.2)		6 (12.2)	2 (4.2)		6 (12.2)	2 (4.2)	
**Lymphatic Invasion** (*n* = 88)										
0	50 (56.8)	17 (38.6)	33 (75.0)	**0.001**	16 (35.6)	34 (79.1)	**< 0.001**	23 (52.3)	27 (61.4)	0.519
1	38 (43.2)	27 (61.4)	11 (25.0)		29 (64.4)	9 (20.9)		21 (47.7)	17 (38.6)	
**Venous Invasion** (*n* = 88)										
0	51 (58.0)	21 (47.7)	30 (68.2)	0.084	19 (42.2)	32 (74.4)	**0.004**	23 (52.3)	28 (63.6)	0.388
1	37 (42.0)	23 (52.3)	14 (31.8)		26 (57.8)	11 (25.6)		21 (47.7)	16 (36.4)	
**Perineural Invasion** (*n* = 88)										
0	73 (83.0)	33 (75.0)	40 (90.9)	0.089	32 (71.1)	41 (95.3)	**0.006**	35 (79.5)	38 (86.4)	0.571
1	15 (17.0)	11 (25.0)	4 (9.1)		13 (28.9)	2 (4.7)		9 (20.5)	6 (13.6)	
**Tumour Budding** (*n* = 87)										
1	46 (52.9)	18 (41.9)	28 (63.6)	**0.041**	19 (42.2)	27 (64.3)	0.063	19 (43.2)	27 (62.8)	0.172
2	17 (19.5)	8 (18.6)	9 (20.5)		9 (20.0)	8 (19.0)		11 (25.0)	6 (14.0)	
3	24 (27.6)	17 (39.5)	7 (15.9)		17 (37.8)	7 (16.7)		14 (31.8)	10 (23.3)	
**TBC (% expanding)** (*n* = 90)										
Mean (SD)	45.0 (29.5)	45.8 (30.7)	44.2 (28.6)	0.827	41.0 (30.4)	49.2 (28.2)	0.12	45.1 (30.8)	44.9 (28.5)	0.936
**Klintrup-Mäkinen** (*n* = 88)										
0	5 (5.7)	4 (9.1)	1 (2.3)	**0.044**	5 (11.1)	0 (0.0)	**< 0.001**	4 (9.1)	1 (2.3)	**0.001**
1	47 (53.4)	28 (63.6)	19 (43.2)		31 (68.9)	16 (37.2)		30 (68.2)	17 (38.6)	
2	31 (35.2)	11 (25.0)	20 (45.5)		9 (20.0)	22 (51.2)		10 (22.7)	21 (47.7)	
3	5 (5.7)	1 (2.3)	4 (9.1)		0 (0.0)	5 (11.6)		0 (0.0)	5 (11.4)

**Table 2:** Clinicopathologic patient characteristics and associations with immune cell density in the TP25μm zone at the tumour centre in cohort 2. Tumour budding is scored according to the 10-high-power-fields method [24]. *Abbreviation: GZMB* granzyme B

		CD8			GZMB			CD68		
	Overall	low (%)	high (%)	p	low (%)	high (%)	p	low (%)	high (%)	p
**n**	136	68	68		68	68		68	68	
**Age** (*n* = 136)										
Mean (SD)	67.8 (14.9)	69.5 (14.5)	66.1 (15.2)	0.186	69.3 (13.7)	66.3 (16.0)	0.237	68.4 (14.9)	67.1 (15.0)	0.614
**Gender** (n = 136)										
Female	59 (43.4)	29 (42.6)	30 (44.1)	1	31 (45.6)	28 (41.2)	0.729	25 (36.8)	34 (50.0)	0.166
Male	77 (56.6)	39 (57.4)	38 (55.9)		37 (54.4)	40 (58.8)		43 (63.2)	34 (50.0)	
**Location** (n = 136)										
Right	67 (49.3)	33 (48.5)	34 (50.0)	1	32 (47.1)	35 (51.5)	0.732	30 (44.1)	37 (54.4)	0.303
Left	69 (50.7)	35 (51.5)	34 (50.0)		36 (52.9)	33 (48.5)		38 (55.9)	31 (45.6)	
**pT** (n = 136)										
3	114 (83.8)	53 (77.9)	61 (89.7)	0.103	52 (76.5)	62 (91.2)	**0.036**	55 (80.9)	59 (86.8)	0.485
4	22 (16.2)	15 (22.1)	7 (10.3)		16 (23.5)	6 (8.8)		13 (19.1)	9 (13.2)	
**Tumour Grade** (n = 136)										
1-2	126 (92.6)	65 (95.6)	61 (89.7)	0.324	66 (97.1)	60 (88.2)	0.1	64 (94.1)	62 (91.2)	0.743
3	10 (7.4)	3 (4.4)	7 (10.3)		2 (2.9)	8 (11.8)		4 (5.9)	6 (8.8)	
**Venous Invasion** (n = 136)										
0	111 (86.7)	54 (85.7)	57 (87.7)	0.945	53 (84.1)	58 (89.2)	0.555	53 (86.9)	58 (86.6)	1
1	17 (13.3)	9 (14.3)	8 (12.3)		10 (15.9)	7 (10.8)		8 (13.1)	9 (13.4)	
**Tumour Budding** (n = 136)										
0	98 (72.1)	48 (70.6)	50 (73.5)	0.848	45 (66.2)	53 (77.9)	0.181	53 (77.9)	45 (66.2)	0.181
1	38 (27.9)	20 (29.4)	18 (26.5)		23 (33.8)	15 (22.1)		15 (22.1)	23 (33.8)	

In cohort 1 we retrospectively included 168 primary CRC patients who received surgical treatment between 2002 and 2013 at the University Hospital of Bern (Switzerland). After excluding 7 patients with missing TNM stage, 3 patients with TNM stage 0, 29 patients who received neoadjuvant chemotherapy and 22 patients who died within 30 days after surgery, a collective of 107 patients remained available for further analysis. GI-expert pathologists (A.L. and H.D.) who were blinded to clinical endpoints re-reviewed cases based on the TNM classification system (7th edition). Overall survival, which was collected from the patients’ clinical records, was defined as the clinical endpoint for cohort 1.

Cohort 2 consisted of a retrospective collective of 177 TNM stage II primary colorectal cancer patients who underwent surgical treatment between 1992 and 2010 at the Mount Sinai University Hospital in Toronto (Canada). 13 patients who died within 6 months after surgery were excluded as regarding their TNM stage II disease this is likely to be a result either from tumour-independent factors or from undisclosed metastases at the time of surgery. Furthermore, 13 patients with a follow-up less than 3 years were also excluded, which left 151 patients available for further analyses. Cases were reviewed by examiners blinded to clinical endpoints based on the TNM classification system (6th edition) and clinical data were collected from patients’ records. For cohort 2, due to the patients’ low-stage disease, disease-free survival was defined as the clinical endpoint.

The use of patient samples was permitted by the ethics commission of the canton of Bern for cohort 1 (KEK 2020-00498) and the research ethics board of the Mount Sinai Hospital, Toronto for cohort 2 (nr13-0136).

### Next-generation tissue microarray construction

Histological analyses were conducted on a tissue microarray (TMA) of formalin-fixed paraffin-embedded tissue using the next-generation tissue microarray (ngTMA®) approach [25]. Annotations were drawn on H&E stained scans of whole slides (P250; 3DHistech, Budapest, Hungary). In cohort 1, annotations were made at four distinct tissue regions for every patient as displayed in Fig. 1a. This included the tumour centre (CT), the tumour front (FR) which was defined as the mainly tumour containing area at the invasive margin, the tumour microenvironment (ME) as the mainly stromal cell containing area at the invasive margin and non-tumorous normal colorectal epithelium (NE). *n* = 2 cores with a diameter of *d* = 0.6 mm were included for every tissue region per patient of cohort 1. Additionally for every tissue region, *n* = 8 cores matching the spatial criteria were included from patients that were not part of the cohort. In cohort 2, annotations were made at the tumour centre (CT) to validate observations from cohort 1. *n* = 2 cores with a diameter of *d* = 1.0 mm were included per patient of cohort 2.

### Triple immunohistochemistry

**Fig. 2:**
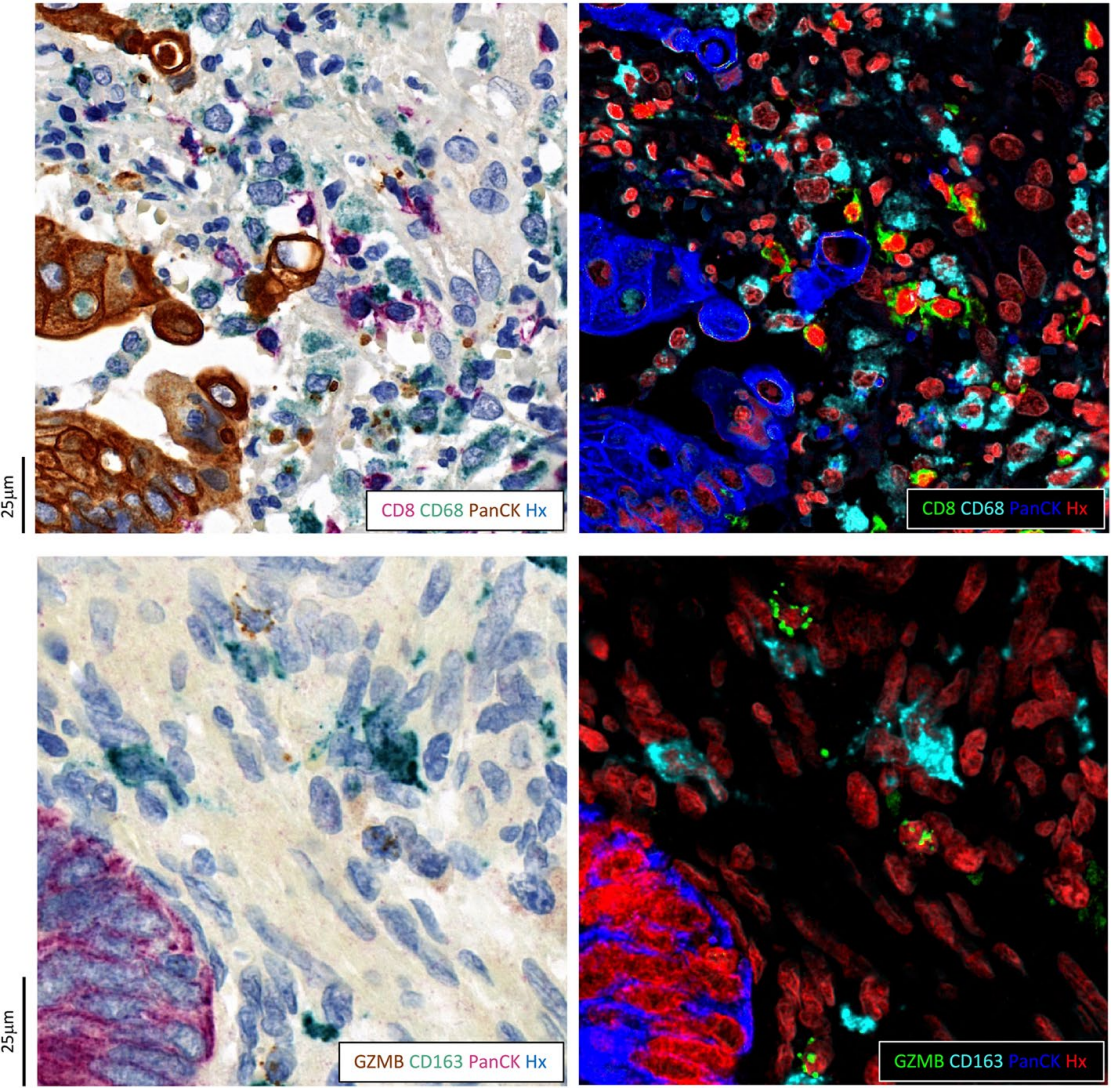
Comparison of brightfield scans (3D Histech, P150) on the left and 4-channel images after colour deconvolution on the right for two triple immunohistochemical stainings. Abbreviations: GZMB, granzyme B; PanCK, pancytokeratin; Hx, haematoxylin

To obtain spatial information about the location of immune cells relative to the tumour, two different IHC stainings were performed on two sequential cuts of all TMA blocks. Pancytokeratin (PanCK) was visualized in both stainings to define tumour expansion and it was complemented with CD8 and CD68 in the first section and with GZMB and CD163 in the second section (Figs. 1b and 2). Combinations and colouring of these markers were chosen based on stain characteristics for optimal visualization. This included combining expectedly non-colocalising markers in a staining because overlapping chromogenic signals result in non-linear amplifications.

IHC staining was performed using an automated system (BOND RX, Leica Biosystems, UK). TMA blocks were cut at 2.5 μm, deparaffinized, and rehydrated in dewax solution (Leica Biosystems). The subsequent steps are schematically presented in Fig. S1: Heat-induced epitope retrieval was performed at pH 9 in Tris buffer (code AR9640, Leica Biosystems) for 30 min at 95 °C. Primary antibodies were incubated sequentially: In the first step, PanCK antibody (Agilent, clone AE/AE3 Ref M3515; dilution 1:400) or GZMB antibody (Biosystems, Clone 11F1, Ref NCL-L-GRAN-B; dilution 1:100) were incubated for 30 min for both stainings respectively. Then all samples were incubated with HRP (Horseradish Peroxidase)-polymer for 15 min and subsequently visualized using 3,3-Diaminobenzidine (DAB) as brown chromogen (Bond polymer refine detection, Leica Biosystems, Ref DS9800) for 10 min. In the second step, CD8 antibody (Dako / Agilent, clone C8/144B, Ref M7103; dilution 1:100) or PanCK antibody (Agilent, clone AE/AE3 Ref M3515; dilution 1:400) were incubated for 8 min or 15 min respectively. Next, secondary antibody AP (Alkaline phosphatase)-polymer was incubated for 8 min and visualized using fast red as a red chromogen (Red polymer refine Detection, Leica Biosystems, Ref DS9390). In the third step, CD68 antibody (Dako / Agilent, clone KP1, Ref M0814; dilution 1:5000) and CD163 antibody (Biosystems, clone 10D6, Ref NCL-CD163; dilution 1:400) were incubated for 15 min for both cuts respectively. Secondary antibody AP (Alkaline phosphatase)-polymer was incubated for 8 min and visualized using PermaGreen Plus as a green chromogen (Diagnostic BioSystems, Ref K959). Finally, the samples were counterstained with haematoxylin and mounted with Aquatex (Merck).

### Digital image analysis

All slides were scanned (P150; 3DHistech, Budapest, Hungary) and TMAs were de-arrayed using open-source software *QuPath* (University of Edinburgh, UK) [26]. Missing, damaged or out-of-focus cores were manually excluded. The brightfield images of the remaining cores were then transformed to 4-channel images with each channel corresponding to one stain using a *Group Sparsity Model* [27] (Fig. 2). Within this model, restriction of one stain per group, λ = 0.1 and non-negativity constraint was applied. These calculations were conducted in Python (v3.9) using the *SPAMS* [28] library (v2.6.1). All subsequent image analyses were performed on the 4-channel images in *QuPath* (Fig. S2).

We used eight TMA cores on each slide of cohort 1 that did not originate from patients included in the cohort to determine the most accurate procedure for our automated analysis. This included a GI-expert pathologist (H.D.) manually setting channel intensity thresholds for automated tissue detection, tumour-stroma differentiation, watershed cell detection and cell type classification as well as supervising training of an object classifier used to exclude artefacts. For the cohort 2 the thresholds had to be manually adapted, correcting for different overall stain intensities. The exact same workflow and thresholds were applied in the automated analysis of all cores within each cohort.

As this study was focusing on peritumoral inflammatory infiltrate, we included TMA cores in further analyses where more than 10 cells were detected within the stroma area and where the tumour area proportion was bigger than 1% of the total tissue area. Cells within the stromal compartment were classified as either CD8^+^, CD68^+^ or double-negative for staining 1 and GZMB^+^, CD163^+^ or double-negative for staining 2. Four tumour proximity (TP) zones were drawn around tumour areas with radii of 10 μm, 25 μm, 50 μm, 100 μm respectively (TP10μm, TP25μm, TP50μm, TP100μm). Density of CD8^+^, GZMB^+^, CD68^+^ and CD163^+^ cells were calculated for each of the four TP zones as well as for the total stroma area. For every defined tissue region (CT, FR, ME, NE), a weighted mean density was calculated for every patient accounting for varying areas between the multiple cores of the same donor patients.

### Statistical analysis

The statistical analyses were conducted using the R statistical programming language (version 4.1.1). We assumed that data were missing at random. Paired *t*-test was applied to determine differences in the log values of immune cell type density close versus distant to the tumour. Correlations between different immune cell types were examined using Pearson’s correlation coefficients of log values of immune cell type densities. As the logarithm of zero is not defined, the cell density (*1/mm*^*2*^) was increased by + 1 for both paired *t*-test and Pearson’s correlation. Associations of nominal and ordinal clinicopathological features with immune cell types were examined using χ^2^ test or Fisher’s exact test where the expected number of observations was smaller than 5 (Histological Subtype, pT, pN, Tumour Grade, Klintrup-Mäkinen all in cohort 1). For scale features *t*-test was applied in case of parametric data (Patient Age) and Kruskal-Wallis test was used for assumedly non-parametric data (Tumour Border Configuration). For univariate survival analyses Kaplan-Meier method and log-rank test was applied except for the comparison of the different spatial regions, where univariate Cox regression model was used. Multivariate survival analyses were conducted using the Cox regression model.

In order to simplify interpretation of hazard ratios (HR), binary groups of immune cell type density (*low* vs. *high*) were formed using the median as a cut-off for single markers. For the combined GZMB/CD68 groups, patients with densities of both immune cell types above the median were classified as *high* and patients with at least one of them below the median as *low*.

## Results

### Frequencies and correlations among immune cells

Densities of CD8^+^, GZMB^+^, CD68^+^ and CD163^+^ cells were calculated for stroma areas at different areas of interest. This included tumour proximity (TP) zones with different radii around the tumour (TP10μm, TP25μm, TP50μm, TP100μm) each for CT, FR, ME and NE (where TP zones were measured from the normal epithelium).

**Fig. 3:**
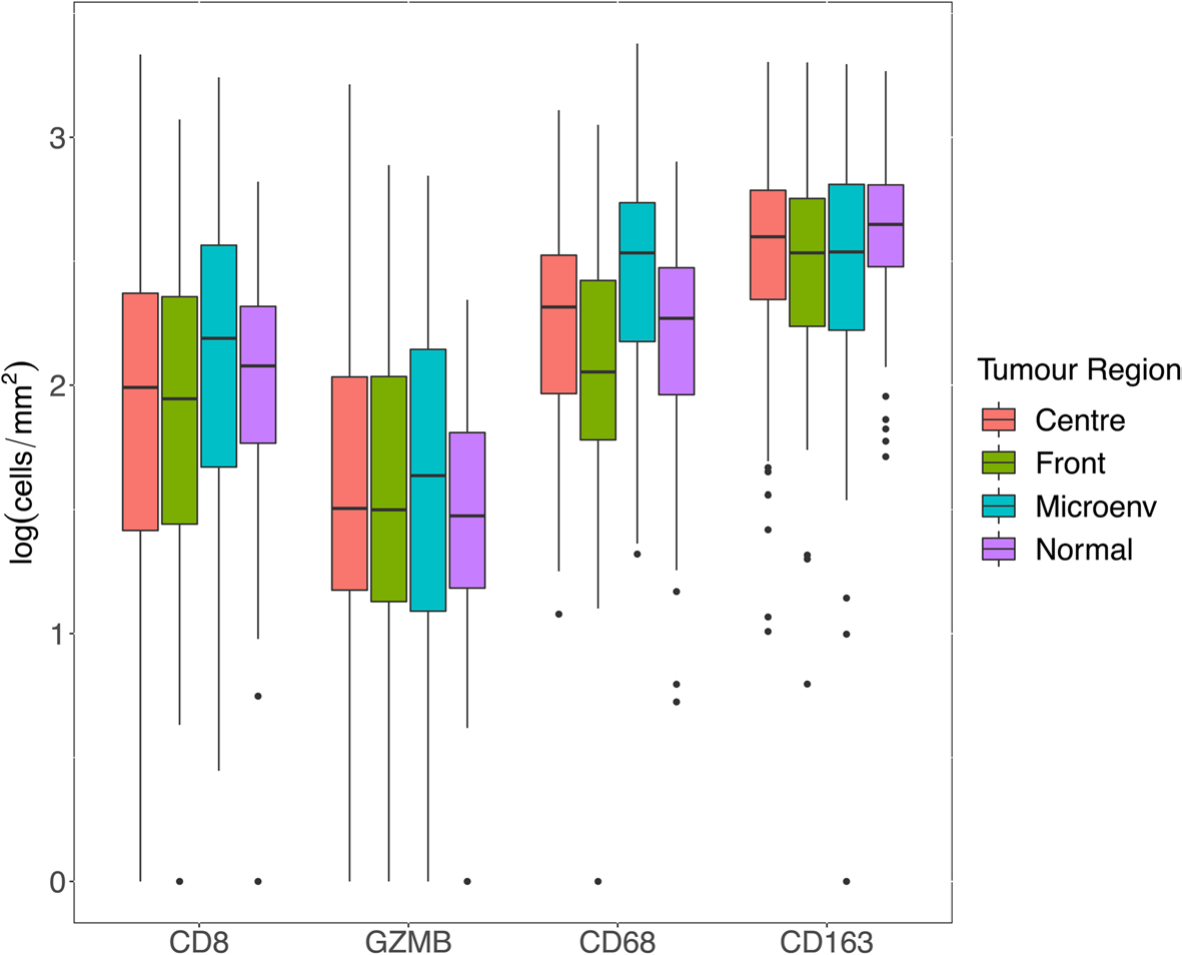
Overall distribution of immune cell density within the stromal compartment for different tumour regions and normal epithelium in cohort 1. Abbreviations: GZMB, granzyme B; Microenv, microenvironment

Distributions of immune cell type densities in the total stroma area for the different tumour regions and normal epithelium are shown in Fig. 3. Comparison of densities between TP25μm zone and the complementary stroma area more distant from the tumour is shown in Fig. S3 for the tumour centre. Whereas the density of GZMB and CD68 did not differ between these areas, CD8 (*p* = 0.003) and CD163 (*p* < 0.001) were significantly lower in the TP25μm zone.

Interrelationships between immune cell types are shown in Table S1. Strong correlations were observed between various immune cell types at different tumour regions, including between all examined immune cell types at the tumour centre (for all *p* < 0.01).

### Survival analysis

Survival data was obtained for all patients in cohort 1 (*n* = 107) and for all patients except one (unclear recurrence status) in cohort 2 (*n* = 150). Overall survival was assessed in cohort 1 and disease-free survival in cohort 2. Median follow-up time was 49 months (range 1-183 months) with occurrence of 33 events in cohort 1 and 65 months (range 45-130 months) with occurrence of 38 events in cohort 2. To achieve maximum comparability between immune cell types, patients with missing data for at least one stained antigen (missing or non-eligible TMA cores) were excluded from survival analyses of the corresponding tissue region. Effect size of immune cell type density on survival was examined in a univariate analysis for different areas of interest in cohort 1 (Table 3) and cohort 2 (Table S2). In cohort 1, the strongest prognostic effects were generally observed at the tumour centre, followed by the tumour front and the tumour microenvironment. Within the tumour centre, comparison of TP zones with varying radii around the tumour revealed the largest number of highly significant associations (*p* < 0.001) for the TP25μm zone. Additionally, the TP25μm zone also showed the lowest mean hazard ratio for CD8, GZMB and CD68 (mean HR = 0.18) compared to the TP10μm zone (mean HR = 0.21) and the TP50μm zone (mean HR = 0.20). CD163 was inferior to the other markers in every region of interest, however significant associations of a high CD163 with longer survival (*p* = 0.012) were still observed in the total stroma area at the tumour centre. As expected, no significant correlations were observed for any immune cell type and TP zone upon examination of the normal colorectal epithelium.

**Table 3:**
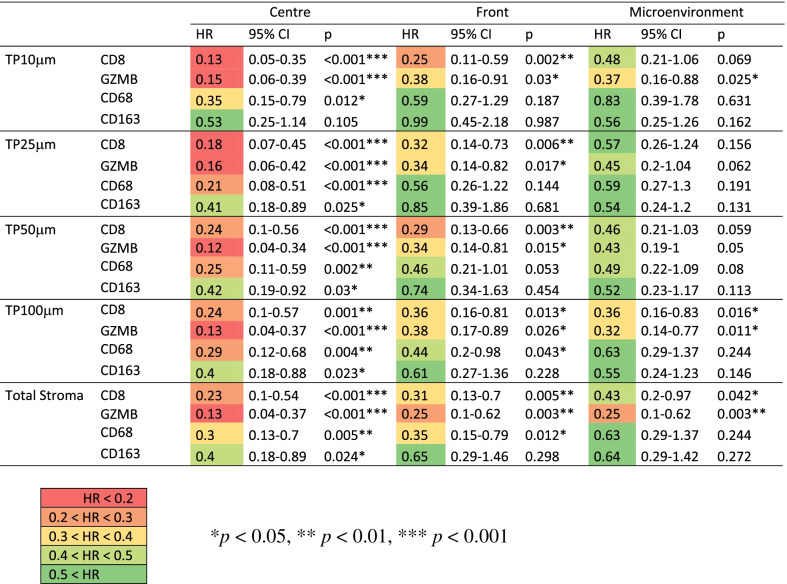
Univariate analysis of overall survival for different regions of interest in cohort 1 (by Cox regression). X-axis (Centre, Front, Microenvironment) indicates annotated tumour regions, Y-axis indicates examined TP zone with various peritumoral radii (10 – 100 μm, Total Stroma: no limitation). *Abbreviations: TP* tumour proximity, *HR* hazard ratio, *CI* confidence interval, *GZMB* granzyme B

Due to these findings, further analyses were based on CD8, GZMB and CD68 densities divided into two groups by the median in the TP25μm zone at the tumour centre. Additionally, patients were allocated to two groups based on the combined GZMB and CD68 density where patients with both densities above the median were classified as GZMB/CD68 *high* and patients with at least one below the median as *low*. This combination was chosen to be compared with the current “benchmark” of the CD8 density and its thoroughly described prognostic effect [3, 11].

**Fig. 4:**
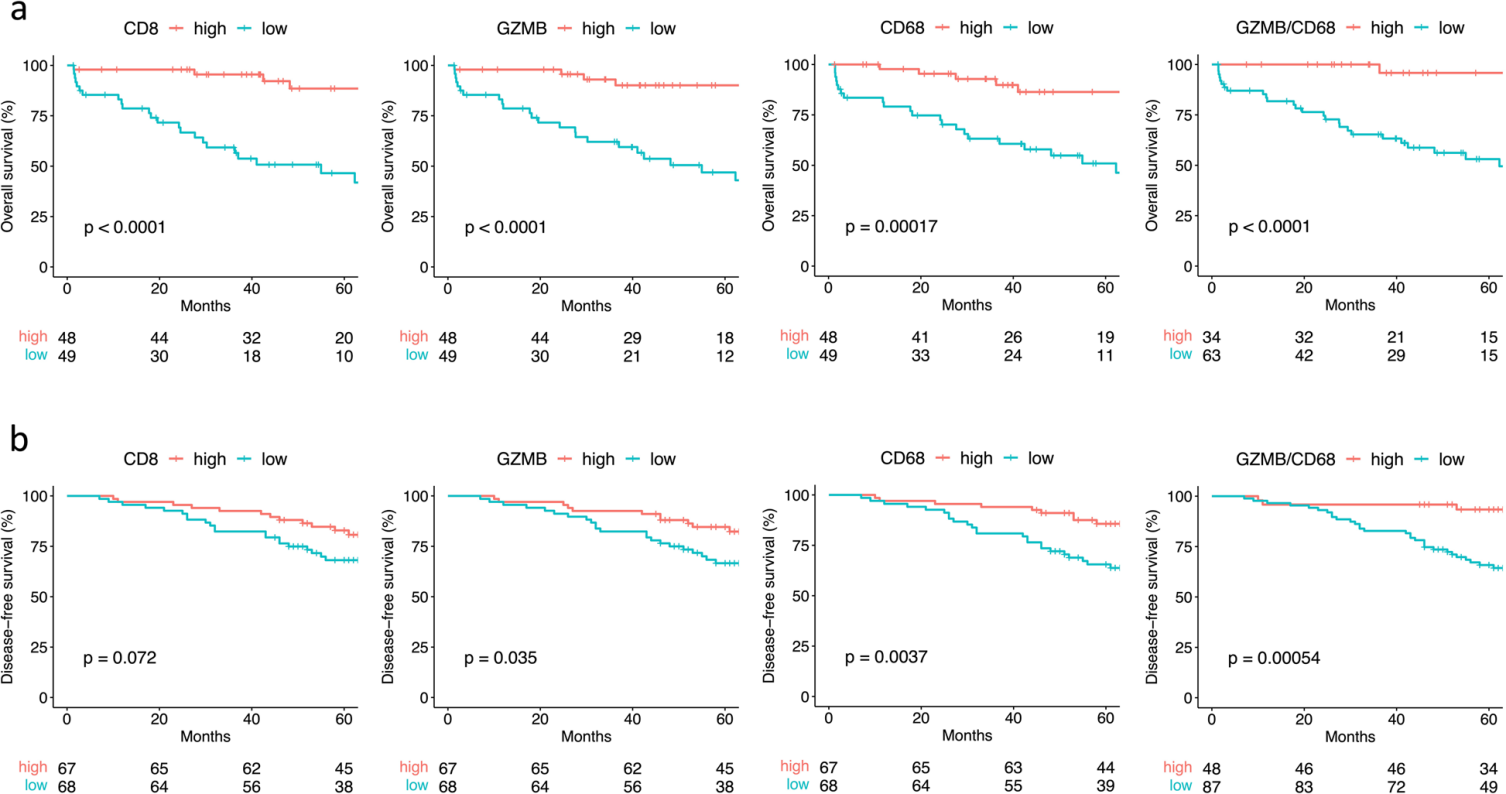
Kaplan-Meier curves for overall and disease-free survival of patients in cohort 1 (**a**) and 2 (**b**) respectively. *P*-values were calculated by the log-rank method. Median density withing the TP25μm zone at the tumour centre was used as cut-off for single marker group formation (CD8, GZMB, CD68). For the combined GZMB/CD68 group formation, patients with GZMB and CD68 densities both above the median were classified as *high* and patients with at least one of them below the median as *low*. Abbreviations: GZMB, granzyme B

Kaplan-Meier curves are displayed in Fig. 4. In cohort 1, highly significant associations (all *p* < 0.001) with longer overall survival were observed for high TP25μm density of CD8, GZMB and CD68 as well as for a high combined GZMB/CD68 score. In cohort 2, longer disease-free survival was associated with high GZMB (*p* = 0.035), high CD68 (*p* = 0.004) and a high combined GZMB/CD68 score (*p* < 0.001). However, CD8, despite a trend, (*p* = 0.072) did not show a significant association with disease-free survival in the TNM stage II cohort. No statistically significant association was observed in any of the two cohorts neither for GZMB:CD8 ratio nor for CD68:CD163 ratio nor for subtracting CD163 from CD68.

In a multivariate survival analysis, prognostic effect of CD8 was compared with the combined GZMB/CD68 score in both cohorts upon inclusion of additional, clinically important variables (Table 4). In the heterogenous cohort 1, the additional variables were TNM stage, the current prognostic gold standard, and administration of adjuvant chemotherapy, an important potential confounder of survival. In cohort 2, consisting of a more homogenous patient population, pT stage and presence of venous invasion were included as additional variables to account for the current basis of clinical decision-making. In cohort 1 both high CD8 (*p* = 0.002) and high combined GZMB/CD68 score (*p* = 0.007) remained significant predictors of longer overall survival with roughly similar hazard ratios upon inclusion of TNM stage and post-operative chemotherapy as additional variables. In cohort 2, prognostic effect on disease-free survival of the combined GZMB/CD68 score (*p* = 0.005) was superior to CD8 (*p* = 0.142) with pT stage and venous invasion as additional variables.

**Table 4:** Multivariate survival analysis comparing prognostic value of CD8 and joint GZMB/CD68 in cohorts 1 and 2 (by Cox regression). Overall survival was used for cohort 1 and disease-free survival for cohort 2. Groups were formed based on immune cell density in the TP25μm zone at the tumour centre. *Abbreviations: HR* hazard ratio, *CI* confidence interval, *GZMB* granzyme B

		Model CD8			Model GZMB/CD68		
		HR	95% CI	p	HR	95% CI	p
Cohort 1 *(TNM I-IV)*	TNM (I-IV)	1.639	1.10-2.43	0.014*	1.556	1.05-2.31	0.028*
	Postoperative Tx (no/yes)	0.62	0.25-1.51	0.292	0.635	0.26-1.57	0.325
	CD8 (low/high)	0.232	0.09-0.58	0.002**			
	GZMB/CD68 (low/high)				0.063	0.01-0.47	0.007**
Cohort 2 *(TNM II)*	pT (3/4)	2.331	1.04-5.22	0.04*	2.165	0.97-4.83	0.059
	Venous invasion (no/yes)	1.119	0.42-2.99	0.822	0.992	0.37-2.65	0.987
	CD8 (low/high)	0.571	0.27-1.21	0.142			
	GZMB/CD68 (low/high)				0.181	0.06-0.60	0.005**

*p < 0.05, **p < 0.01

### Correlation of immune cells with clinicopathological features

Once again by using the median density in the TP25μm zone at the tumour centre as the cut-off, patients were classified as either *high* or *low* for CD8, GZMB and CD68 respectively.

Overall distributions of patients’ clinicopathological features and their associations with immune cell density in the TP25μm zone at the tumour centre are displayed for both cohorts in Tables 1 and 2. In cohort 1, notably high CD8, high GZMB and high CD68 were significantly associated with lower TNM stage and higher Klintrup-Mäkinen score. GZMB was the sole variable that showed significant associations of high density with lower pT stage (*p* = 0.036), absent venous (*p* = 0.004) and absent perineural invasion (*p* = 0.006). High CD8 was the only variable significantly associated with less tumour budding (*p* = 0.041). In cohort 2, the only significant association was observed between high GZMB and lower pT stage (*p* = 0.036).

## Discussion

The novel findings of this study include the independent positive prognostic effect of a high combined peritumoral GZMB^+^ and CD68^+^ cell density in a primary cohort (TNM stage I-IV) and in a validation cohort (TNM stage II) in CRC patients. Based on multiplex IHC, a combined GZMB/CD68 score demonstrated a stronger prognostic effect than CD8^+^ cell density alone in TNM stage II patients in a multivariate analysis. This indicates that the mere presence of CD8^+^ T lymphocytes, which itself is an established component of previously proposed scores to assess the host’s immune reaction against the tumour [3, 11], might not sufficiently represent the actual potency of the antitumoral cytotoxic function. Instead, downstream indicators of effective tumour recognition and immune effector function, such as activated CD8^+^ cells expressing the protease GZMB inducing apoptosis in the target cell or ongoing tumour cell phagocytosis by CD68^+^ macrophages might be of more value in determining the prognosis of CRC patients.

The distribution of immune cell density showed a wider interquartile range (IQR) for all tumour regions (CT, FR, ME) than in the normal colorectal epithelium for all assessed antigens (CD8, GZMB, CD68, CD163). This increased variation can be interpreted as a consequence of tumour cells either being recognized as such by the immune system, and thus facing an inflammatory response, or as tumour cells preventing an efficient immune reaction by checkpoint inhibition, which has been described extensively in the past [29, 30].

CD8^+^, GZMB^+^, CD68^+^ and CD163^+^ cells showed significant correlation throughout the majority of assessed tumour regions (centre, front and microenvironment). This finding was mirrored by several strong associations between high density of these markers with less aggressive clinicopathological characteristics and improved overall survival in cohort 1. Interestingly, only one statistically significant association was observed between immune cell density and clinicopathological features (GZMB-pT stage) despite trends (CD8) and significant correlations (GZMB, CD68) with disease-free survival in the TNM stage II patients of cohort 2. This highlights the unsatisfactory situation of the currently available prognostic parameters for patients with stage II disease.

Previous research has shown associations of a peritumoral infiltrate rich in CD8^+^ cells with longer survival in colorectal cancer, which led to its inclusion in the Immunoscore [11, 13]. The results of this study basically support these findings with an observed strong prognostic effect for CD8^+^ cells in cohort 1, however only a trend towards longer disease-free survival was seen in cohort 2. As a contrast to the abundantly examined CD8, GZMB has only occasionally caught attention as a predictor of positive outcome in the past [21, 22]. This may also be a consequence of the sparse and granular expression of GZMB which makes manual scoring a challenging task. In this study, automated digital image analysis presented an efficient and unbiased way of GZMB assessment. Our results demonstrated equal to slightly better prognostic characteristics of GZMB compared to CD8. In the context of immune-checkpoint inhibition [31] and immunoediting [32] this presents an indication that different states of activation might be present among CD8^+^ cells with only a fraction displaying activated cytotoxic characteristics, such as expression of GZMB. It also must be considered that GZMB expression is not limited to CD8^+^ T cells alone and that especially natural killer (NK) cells, despite their sparse occurrence, could have contributed to the GZMB^+^ cell count. Previous research has produced contradictory results regarding prognostic significance of CD68^+^ and CD163^+^ tumour associated macrophages (TAMs), reporting both negative [14] and positive [8, 15, 16] prognostic effect. The results of this study matched recent research [20] by underlining that differentiation of macrophage phenotypes is crucial as major differences were observed between prognostic impact of CD68 and CD163 density in both cohorts. Discordant with previous work examining macrophage subset ratios [10, 19, 20], CD68:CD163 ratio was not associated with survival in our cohorts. From a mechanistical point of view, M1 polarized macrophages can be seen as active removers of both apoptotic (i.e. targeted by cytotoxic lymphocytes) and opsonized tumour cells (via Fc dependent phagocytosis). Thus, presence of M1-like macrophages might integrate both an effective cellular and humoral antitumoral immunity with only relatively few studies having taken the latter into consideration to date [33].

The spatial analyses of this study showed that the tumour centre presented the highest prognostic power of immune cell type density compared with the tumour front and the tumour microenvironment. A strength of this study is the systematic approach of examining multiple TP zones with varying distance to the tumour. Even though previous studies have considered the spatial positions of inflammatory cells relative to the tumour [10, 11], it remains unclear how their spatial zones were established. While Nearchou et al. [10] calculated immune cell densities within an area with a radius of 50 μm around tumour cells, the Immunoscore’s [11] “invasive margin” measurement is based on an area with a diameter of 1000 μm. Regarding the prognostic value of tumour proximity (TP) zones with varying radii around the tumour in this study, only a weak trend could be observed with the TP25μm zone presenting the lowest hazard ratios in both cohorts. The only minor differences might be a consequence of the study design based on TMAs where a spatial selection had already been applied before and only allowed analyses of small tissue extracts. Interestingly, density of CD8^+^ and GZMB^+^ cells were of consistent prognostic value regardless of their tumour proximity, whereas CD68^+^ cell density had the greatest prognostic effect in the TP25μm zone and decreased in locations more distant to the tumour. This pattern was be observed at the tumour centre in both cohorts and underlines current opinion that macrophages might play complementary roles in the tumour environment with tumour-adjacent macrophages in particular displaying antitumoral phagocytic activities. The weaker prognostic effect in the TP10μm zone might be as a result of tumour retraction artefacts distorting density calculations.

Key limitations of this study include limited tissue areas available for examination due to the TMA approach, missing patients’ mismatch-repair (MMR) status and potentially biased CD68^+^ and CD163^+^ cell numbers due to difficult cytoplasm-to-nucleus allocation because of numerous cytoplasmatic protrusions. Additionally, due to the lack of a precise M1 macrophage marker, this distinct population could only be estimated indirectly via the pan-macrophages marker CD68 and the M2 macrophage specific CD163. Eventually, the limited number of patients in both cohorts prevented determination of optimum thresholds based on ROC analysis. Thus, effect sizes observed in this study might have been underestimated using the crude measure of the median as a cut-off point.

## Conclusions

This study identified that expression of CD8, GZMB and CD68 within 25 μm of tumour cells at the tumour centre were strongly predictive of survival. In this area, joint high expression of GZMB and CD68 was found to be more strongly associated with improved survival than lone high expression of CD8 in TNM stage II patients. Further research is needed to resolve unclear prognostic effects of macrophages, particularly regarding polarization and spatial occurrence. It should also be confirmed whether the degree of activation in the peritumoral inflammatory infiltrate is not only prognostic, but also predictive of a CRC patient’s response to adjuvant chemotherapy.

## Supplementary Information

**Additional file 1.**

**Additional file 2.**

**Additional file 3.**

**Additional file 4.**

**Additional file 5.**

## Data Availability

The data that support the findings of this study are available from the University of Bern and the Mount Sinai Hospital, but restrictions apply to the availability of these data, which were used under license for the current study, and so are not publicly available. Data are however available from the authors upon reasonable request and with permission of the University of Bern and the Mount Sinai Hospital, Toronto.

## Abbreviations

AP: Alkaline phosphatase
CRC: Colorectal cancer
CT: Tumour centre
DAB: Diaminobenzidine
FR: Tumour front
GZMB: Granzyme B
HR: Hazard ratio
H&E: Haematoxylin and eosin
HRP: Horseradish peroxidase
IHC: Immunohistochemistry
ME: Tumour microenvironment
MMR: Mismatch repair
NE: Normal epithelium
NK: Natural killer
PanCK: Pancytokeratin
TAM: Tumour associated macrophage
TIL: Tumour infiltrating lymphocyte
TMA: Tissue microarray
TNM: Tumour-node-metastasis
TP: Tumour proximity
