# Supplementary material for: A combined spatial score of granzyme B and CD68 surpasses CD8 as an independent prognostic factor in TNM stage II colorectal cancer
Source: BMC Cancer. 2022 Sep 16;22:987. doi: 10.1186/s12885-022-10048-x (PMC9482175; doi:10.1186/s12885-022-10048-x)
Supplement: Supplementary file 1 — Additional file 1. [file 12885_2022_10048_MOESM1_ESM.docx]

**Figure S1**





*Figure S1.* Workflow of the multiplex immunohistochemistry for the two applied stainings. Abbreviations: PanCK, pancytokeratin; GZMB, granzyme B; HRP, horseradish peroxidase; AP, alkaline phosphatase; DAB, diaminobenzidine.
