# Supplementary material for: A combined spatial score of granzyme B and CD68 surpasses CD8 as an independent prognostic factor in TNM stage II colorectal cancer
Source: BMC Cancer. 2022 Sep 16;22:987. doi: 10.1186/s12885-022-10048-x (PMC9482175; doi:10.1186/s12885-022-10048-x)
Supplement: Supplementary file 2 — Additional file 2. [file 12885_2022_10048_MOESM2_ESM.docx]

**Figure S2**





*Figure S2.* Illustration of the digital image analysis workflow. All procedures were performed in *QuPath [26]* based on 4-channel images. Thresholds were defined manually by a pathologist based on tissue that was not part of the cohort. Abbreviations: PanCK, pancytokeratin; GZMB, granzyme B; TP, tumour proximity.
