# Supplementary material for: A combined spatial score of granzyme B and CD68 surpasses CD8 as an independent prognostic factor in TNM stage II colorectal cancer
Source: BMC Cancer. 2022 Sep 16;22:987. doi: 10.1186/s12885-022-10048-x (PMC9482175; doi:10.1186/s12885-022-10048-x)
Supplement: Supplementary file 3 — Additional file 3. [file 12885_2022_10048_MOESM3_ESM.docx]

**Figure S3**





*Figure S3.* Comparing distribution of immune cell density at the tumour centre for the TP25μm zone (red) and the tumour-distant area (blue) in cohort 1. Paired *t*-test was used to determine differences within one immune cell type (***p* < 0.01, ****p* < 0.001, n.s.: *p* > 0.05). Abbreviations: GZMB, granzyme B.
