# Supplementary material for: A combined spatial score of granzyme B and CD68 surpasses CD8 as an independent prognostic factor in TNM stage II colorectal cancer
Source: BMC Cancer. 2022 Sep 16;22:987. doi: 10.1186/s12885-022-10048-x (PMC9482175; doi:10.1186/s12885-022-10048-x)
Supplement: Supplementary file 4 — Additional file 4. [file 12885_2022_10048_MOESM4_ESM.docx]

**Table S1**

|  | CD8_CT_ | CD8_FR_ | CD8_ME_ | GZMB _CT_ | GZMB _FR_ | GZMB _ME_ | CD68 _CT_ | CD68 _FR_ | CD68 _ME_ | CD163 _CT_ | CD163 _FR_ |
| --- | --- | --- | --- | --- | --- | --- | --- | --- | --- | --- | --- |
| CD8 _FR_ | 0.751*** | - | - | - | - | - | - | - | - | - | - |
| CD8 _ME_ | 0.661*** | 0.634*** | - | - | - | - | - | - | - | - | - |
| GZMB _CT_ | 0.733*** | 0.535*** | 0.575*** | - | - | - | - | - | - | - | - |
| GZMB _FR_ | 0.630*** | 0.767*** | 0.631*** | 0.623*** | - | - | - | - | - | - | - |
| GZMB _ME_ | 0.551*** | 0.538*** | 0.789*** | 0.602*** | 0.707*** | - | - | - | - | - | - |
| CD68 _CT_ | 0.559*** | 0.349** | 0.500*** | 0.550*** | 0.410*** | 0.342** | - | - | - | - | - |
| CD68 _FR_ | 0.414*** | 0.579*** | 0.420*** | 0.487*** | 0.587*** | 0.415*** | 0.568*** | - | - | - | - |
| CD68 _ME_ | 0.423*** | 0.199 | 0.576*** | 0.476*** | 0.346** | 0.591*** | 0.516*** | 0.480*** | - | - | - |
| CD163 _CT_ | 0.581*** | 0.347** | 0.457*** | 0.618*** | 0.309** | 0.324** | 0.679*** | 0.366*** | 0.348** | - | - |
| CD163 _FR_ | 0.535*** | 0.631*** | 0.523*** | 0.350** | 0.584*** | 0.515*** | 0.321** | 0.574*** | 0.247* | 0.588*** | - |
| CD163 _ME_ | 0.457*** | 0.339** | 0.620*** | 0.413*** | 0.356** | 0.591*** | 0.378*** | 0.213 | 0.544*** | 0.632*** | 0.604*** |

*Table S1.* Pearson’s correlations between immune cell type densities in the total stroma area at the tumour centre (CT), front (FR) and microenvironment (ME). *r* values are presented. (**p* < 0.05, ***p* < 0.01, ****p* < 0.001). Abbreviations: GZMB, granzyme B.
