# Supplementary material for: A combined spatial score of granzyme B and CD68 surpasses CD8 as an independent prognostic factor in TNM stage II colorectal cancer
Source: BMC Cancer. 2022 Sep 16;22:987. doi: 10.1186/s12885-022-10048-x (PMC9482175; doi:10.1186/s12885-022-10048-x)
Supplement: Supplementary file 5 — Additional file 5. [file 12885_2022_10048_MOESM5_ESM.docx]

**Table S2**

|  |  | Centre | | |
| --- | --- | --- | --- | --- |
|  |  | HR | 95% CI | p |
| TP10μm | CD8 | 0.56 | 0.29-1.1 | 0.092 |
|  | GZMB | 0.4 | 0.19-0.84 | 0.016* |
|  | CD68 | 0.45 | 0.23-0.9 | 0.024* |
|  | CD163 | 0.63 | 0.31-1.26 | 0.192 |
| TP25μm | CD8 | 0.5 | 0.25-0.98 | 0.042* |
|  | GZMB | 0.47 | 0.23-0.96 | 0.039* |
|  | CD68 | 0.33 | 0.16-0.68 | 0.003** |
|  | CD163 | 0.63 | 0.32-1.28 | 0.201 |
| TP50μm | CD8 | 0.49 | 0.25-0.97 | 0.041* |
|  | GZMB | 0.4 | 0.19-0.84 | 0.016* |
|  | CD68 | 0.45 | 0.23-0.91 | 0.025* |
|  | CD163 | 0.54 | 0.27-1.11 | 0.092 |
| TP100μm | CD8 | 0.49 | 0.25-0.97 | 0.041* |
|  | GZMB | 0.4 | 0.19-0.83 | 0.014* |
|  | CD68 | 0.53 | 0.27-1.04 | 0.064 |
|  | CD163 | 0.54 | 0.27-1.11 | 0.093 |
| Total Stroma | CD8 | 0.49 | 0.25-0.97 | 0.041* |
|  | GZMB | 0.39 | 0.19-0.83 | 0.014* |
|  | CD68 | 0.61 | 0.31-1.19 | 0.146 |
|  | CD163 | 0.64 | 0.32-1.29 | 0.209 |

| HR < 0.2 |
| --- |
| 0.2 < HR < 0.3 |
| 0.3 < HR < 0.4 |
| 0.4 < HR < 0.5 |
| 0.5 < HR |

*Table S2.* Univariate analysis of disease-free survival for TP zones with different radii (10 – 100 μm, Total Stroma: no limitation) in cohort 2 (by Cox regression). Abbreviations: TP, tumour proximity; HR, hazard ratio; CI, confidence interval; GZMB, granzyme B.
